# Photoinduced Endosomal Escape Mechanism: A View from Photochemical Internalization Mediated by CPP-Photosensitizer Conjugates

**DOI:** 10.3390/molecules26010036

**Published:** 2020-12-23

**Authors:** Tet Htut Soe, Kazunori Watanabe, Takashi Ohtsuki

**Affiliations:** 1 Department of Biotechnology, Mandalay Technological University, Patheingyi, Mandalay 05072, Myanmar; tethtutsoe@mtu.edu.mm; 2 Department of Interdisciplinary Science and Engineering in Health Systems, Okayama University, 3-1-1 Tsushimanaka, Okayama 700-8530, Japan; k_watanabe@okayama-u.ac.jp

**Keywords:** photochemical internalization, photosensitizer, cell-penetrating peptide, endosome, membrane

## Abstract

Endosomal escape in cell-penetrating peptide (CPP)-based drug/macromolecule delivery systems is frequently insufficient. The CPP-fused molecules tend to remain trapped inside endosomes and end up being degraded rather than delivered into the cytosol. One of the methods for endosomal escape of CPP-fused molecules is photochemical internalization (PCI), which is based on the use of light and a photosensitizer and relies on photoinduced endosomal membrane destabilization to release the cargo molecule. Currently, it remains unclear how this delivery strategy behaves after photostimulation. Recent findings, including our studies using CPP-cargo-photosensitizer conjugates, have shed light on the photoinduced endosomal escape mechanism. In this review, we discuss the structural design of CPP-photosensitizer and CPP-cargo-photosensitizer conjugates, and the PCI mechanism underlying their application.

## 1. Introduction

Peptide-based molecular delivery systems employing cell-penetrating peptides (CPPs) have been used for intracellular delivery of cargo molecules, including drugs, peptides/proteins, and nucleic acids. The CPP-based system is useful for therapeutic and diagnostic purposes because of elevated cell permeability and low cytotoxicity of CPPs [1,2]. For therapeutic purposes, delivery of chemotherapeutic agents, nucleic acids, therapeutic proteins, and vaccine peptides has been facilitated through their conjugation to CPPs. In the case of chemotherapeutic delivery, CPPs increase the cellular uptake of chemotherapeutic agents, such as doxorubicin, in cancer cells [3]. In addition to cancer treatment, conjugation of insulin to Tat improves the bioavailability of insulin [2]. Another biomedical application of CPP is the delivery of imaging agents, such as fluorescent quantum dots used for single molecular imaging in living cells [4].

The various CPPs that have been found or developed are divided into three major classes: cationic, amphipathic, and hydrophobic CPPs. Cationic CPPs include natural peptides, such as Tat and antennapedia-homeodomain-derived Antp, as well as synthetic peptides, such as polyarginine (R9) or polylysine (K9) [5,6]. Some widely used amphipathic CPPs originate from naturally occurring peptides; they include the VP22 peptide from the herpes simplex virus VP22 protein, and the MPG peptide generated from the fusion of natural SV40 nuclear localization signal peptide and a viral hydrophobic domain derived from the HIV-gp-41 segment. Hydrophobic CPPs are not employed as commonly as cationic or amphipathic CPPs [5,6]. Arginine-rich CPPs are the most popular because of their cationic properties, which allow for strong binding to the anionic membrane of cells [7]. The interaction between cationic CPPs and negatively charged glycosaminoglycans on the cell surface is thought to be the first step in cellular uptake of CPPs [8].

Cellular uptake of CPPs occurs either through direct membrane translocation or via endocytosis [2]. The mode of cellular uptake of CPP is affected by various factors, such as the nature and concentration of CPPs, size and type of cargo, and membrane composition [8,9,10]. Energy-independent, direct penetration pathways may include several mechanisms that have been reported such as the inverted micelle formation model, pore formation model, carpetlike model, and membrane thinning model [9,11]. The major entry route of CPPs and CPP-cargo conjugates is endocytosis, such as macropinocytosis, clathrin-mediated endocytosis, and caveolin-mediated endocytosis [11]. Different endocytic pathways can be utilized by the same CPP. For example, Tat peptides enter cells via macropinocytosis, whereas Tat-fusion peptides enter cells via lipid-raft-dependent endocytosis [1,12]. For the primary amphipathic CPPs, direct penetration is most probable at high CPP concentrations [9]. CPPs with small cargos exploit direct entry routes at 4 °C, but at 37 °C, endocytosis is a particularly frequent pathway for CPPs carrying macromolecular cargo [10,13,14].

CPP conjugates have been widely studied for biological and clinical applications [2]; however, cellular internalization of CPP-fused molecules via endocytosis often leads to their endosomal entrapment [15,16,17]. A variety of approaches have been developed for the endosomal escape of CPP-cargo conjugates. The endosomolytic activity of CPPs is increased by the use of multivalent CPPs, such as dendrimers and loligomers [5]. Endosomal escape of CPP-cargo conjugates can be increased by attaching pH-dependent membrane-active peptides or fusogenic peptides (e.g., hemagglutinin HA2 peptide) that disrupt membranes at acidic pH [5]. The addition of polyethyleneimine (PEI) to arginine-rich CPP through polyethylene glycol (PEG) linker enhanced the transfection efficiency both in vivo and in vitro through PEI-mediated osmotic lysis of endosomes, which is called the proton sponge effect [18]. Chemical agents, such as chloroquine and Ca^2+^, can enhance the delivery of CPP-cargo molecules [19,20,21], although these chemicals may only have limited uses in vivo because of their cytotoxicity or rapid efflux. Recently, it has been reported that a small molecule, UNC7938, enhances CPP-mediated cytosolic delivery of macromolecules, whereby UNC7939 helps to destabilize the endosomal membrane [22].

Photochemical internalization (PCI) enables the release of endosome-entrapped molecules, such as drugs and biomacromolecules, in the cytoplasm of target cells using a photosensitizer and light as triggers [23,24]. Reactive oxygen species (ROS) photogenerated from the endocytosed photosensitizer are believed to cause peroxidation of endosomal membrane molecules [24,25], which become destabilized or leaky, thus allowing the release of endocytosed molecules [26]. Recently, PCI technology has been developed for cancer treatment and vaccination purposes [27,28,29].

A number of studies have shown that the use of CPP–photosensitizer (CPP-PS) conjugates offers a promising way to deliver macromolecules into cells [30,31,32]. PCI mediated by CPP-PS conjugates is an efficient tool for transducing transient high-cytosolic concentrations of nucleic acids and proteins/peptides in a short duration [31,33]. Optimal use of CPP-PS conjugates for therapeutic delivery depends on efficient membrane destabilization induced by photogenerated ROS and minimal photoinduced damage to cells, except for target endosomes. In this review, we discuss the photoinduced endosomal escape mechanism of CPP-PS and CPP-cargo-PS conjugates.

## 2. PCI Using CPP(-Cargo)-PS Conjugates

PCI using CPP-cargo-PS conjugates offers a promising approach for the phototriggered spatiotemporal release of cargo from endosomes. Another strategy to enhance endosomal escape of CPP-cargo complexes involves the use of endosomolytic CPPs; however, it does not allow for controlled release of cargo [34].

**Figure 1:**
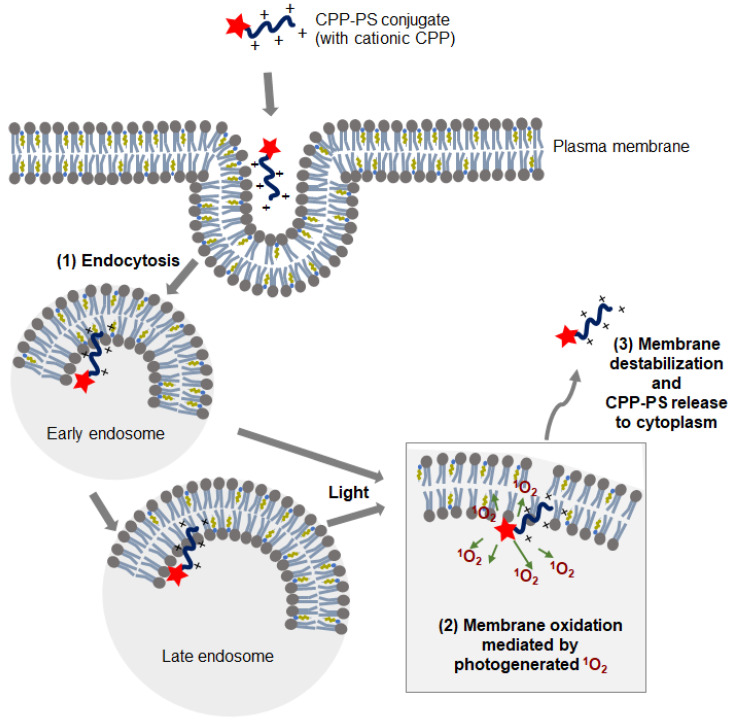
Mechanism of cell-penetrating peptide (CPP)-photosensitizer (PS)-mediated photochemical internalization (PCI). (**1**) The CPP-PS conjugate enters the cell via endocytosis. Due to the cationic nature of CPP, CPP-PS binds to the anionic membrane surface. (**2**) After light irradiation, the photoexcited PS of the conjugate generates reactive oxygen species (ROS), mainly singlet oxygen (^1^O_2_), which causes oxidation of the endosomal membrane. (**3**) Oxidation causes membrane destabilization, leading leaky endosomes to release the CPP-PS conjugate to the cytosol.

When using cationic CPPs, CPP-cargo-PS conjugates tend to be taken up by cells through endocytosis and remain in the endosomal compartment until light irradiation [35]. Probably, the CPP-cargo-PS attaches to the endosomal membrane due to the cationic nature of the CPP. In the presence of a photosensitizer, light promotes the generation of ROS; specifically, singlet oxygen (^1^O_2_). The photogenerated ^1^O_2_ induces membrane oxidation, leading to destabilization of the endosomal membrane and cytosolic release of the conjugate (Figure 1). CPP likely enhances the photooxidation of endosomal membrane molecules, as a photosensitizer conjugated with an arginine-rich peptide was shown to be a more-efficient photolytic membrane agent than a free photosensitizer [36,37,38].

In the following sections, we describe the photosensitizers for PCI (2.1), peptide-PS conjugation methods (2.2), and designs of CPP-cargo-PS conjugates (2.3).

### 2.1. Photosensitizers for PCI

**Figure 2:**
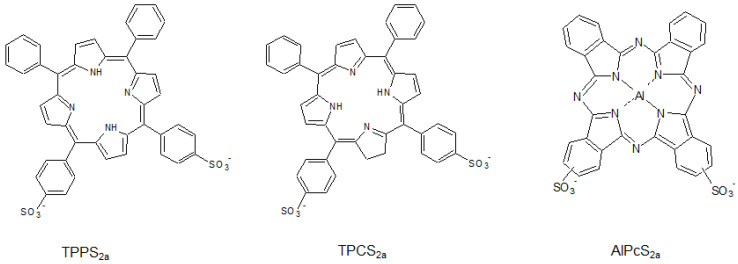
Molecular structures of the commonly used photosensitizers for PCI. Due to their endolysosomal localization ability, they are normally used without conjugation to endolysosomal targeting agents (e.g., CPP).

Many photosensitizers have been developed for photodynamic therapy (PDT), whereby photogenerated ROS mediate destruction of target cells. Some photosensitizers commonly used for PDT can be applied to PCI, as long as they can be localized to the endolysosomal compartment. Although hydrophilic photosensitizers such as tetra(4-sulfonatophenyl)porphine (TPPS_4_) and chlorin p6 derivatives [39,40] can localize to the endo-lysosomal compartment, some studies have suggested that amphiphilic photosensitizers achieve greater PCI efficiency because they enter cells via endocytosis and localize to the lipid–aqueous interface of the membrane [41]. Widely used amphiphilic photosensitizers for PCI include meso-tetraphenyl porphyrindisulphonate (TPPS_2a_), disulfonated aluminum phthalocyanine (AlPcS_2a_), and disulfonatedtetraphenyl chlorin (TPCS_2a_) (Figure 2). Among these photosensitizers, TPCS_2a_ was shown to improve membrane permeability and, hence, was more suitable for PCI in both in vitro and in vivo applications [40,42].

**Figure 3:**
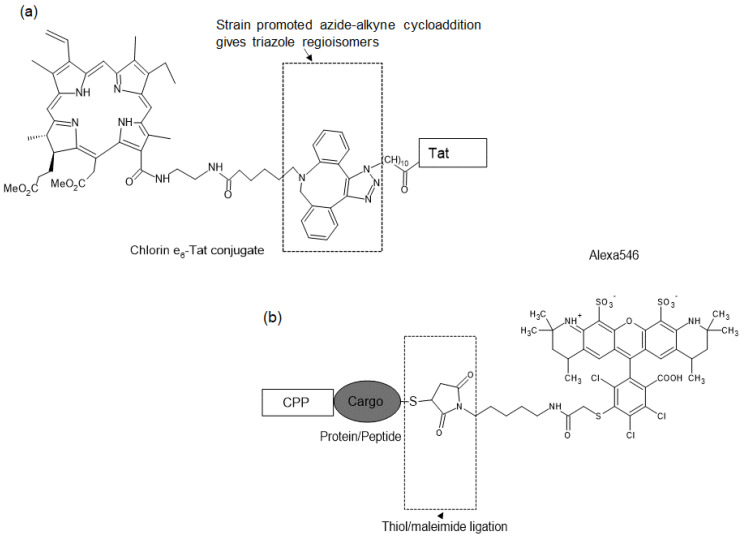
Representative examples of CPP-PS and CPP-cargo-PS conjugates. (**a**) Structural design of CPP-PS [43]. (**b**) CPP-cargo-PS conjugate prepared by our group [16].

Conjugation with CPP (Tat) allows even a photosensitizer such as chlorin e6 (Ce6) that localizes poorly to endosomes to be applied for PCI (Figure 3a) [43]. In the case of some peptide-PS conjugates, the structure and charge of both peptide and photosensitizer have been suggested to affect their cellular uptake and subcellular localization [44,45]. For instance, Tat–polyethylene glycol (PEG)-linked hydrophilic porphyrin was slowly taken up by cells and localized to lysosomes, whereas hydrophobic porphyrin conjugated with the same Tat–PEG showed more cellular accumulation and localization in the endoplasmic reticulum (ER). While there are numerous studies on peptide-PS conjugates for PDT [46], their potential for PCI has been less well characterized.

### 2.2. Peptide-PS Conjugation Methods

The use of peptide-photosensitizer conjugates in both PDT and PCI aims to enhance the photobiological ability of photosensitizers by coupling it to the ability of the peptide to target specific cellular or subcellular locations. To construct efficient peptide-PS conjugates, the conjugation reaction is tailored to the structural characteristics of both the peptide and photosensitizer. Bioconjugation techniques, such as ligation reactions via thiol- and amino-reactive photosensitizers, have been used for the synthesis of peptide-PS conjugates [47,48]. Here, we highlight some important aspects of conjugation required to build functional CPP/peptide-PS conjugates.

Generally, peptides can be linked with photosensitizers by two methods: noncovalent or covalent conjugation. Noncovalent conjugation methods are based on the coassembly of peptides and photosensitizers by simple mixing. For example, nanoparticles for PDT could be prepared by coassembly of a dipeptide with Ce6 [49]. Photocatalytically active peptide–porphyrin microspheres can be assembled using dipeptides (e.g., diphenylalanine) and sulfonated porphyrin [50]. Tat peptide noncovalently conjugated with sulfonated aluminum phthalocyanine (AlPcS) by simple mixing (AlPcS and Tat molar ratio = 1:10) was shown to enhance uptake of the photosensitizer through endocytosis [51].

Covalent ligation offers a much wider choice regarding the nature of the conjugation partner, and there are various techniques for peptide–photosensitizer conjugation [48]. To achieve efficient conjugation, a single reactive group is required for peptides. For instance, a cysteine residue is essential to react with the maleimide group of the photosensitizer. A free terminal amino group or a specifically deprotected side chain amino group in a peptide, in which all side chain amino groups except one are protected, is required for conjugation to amine-reactive photosensitizers during solid-phase synthesis [47]. A study of various synthesis approaches aimed at linking porphyrin derivatives with cationic CPPs (Tat, penetratin, and pVEC) showed that strain-promoted azide-alkyne ligation was more suitable than thiol–maleimide reaction, oxime ligation, or copper-catalyzed azide-alkyne cycloaddition [52]. A CPP-PS conjugate synthesized via strain-promoted azide-alkyne ligation is presented in Figure 3a.

Linker amino acids between the photosensitizer and peptide can improve PCI efficiency of CPP-PS conjugates. LL (LeuLeu) and FF (PhePhe) linkers near the photosensitizer eosin or Alexa Fluor 546 (Alexa546) enhanced the PCI efficacy of peptide-PS conjugates [53]. This is probably because linker amino acids affect the photoreactivity and/or cellular localization of the photosensitizer. The same is also assumed for all functional groups on the photosensitizer. For example, diamino acid modifications such as diaspartate and aspartate-lysine affected cellular localization and phototoxicity of the modified Ce6 [54]. In addition, chemical modifications have been shown to affect the efficiency of ^1^O_2_ photogeneration by modified porphyrins (e.g., hematoporphyrin VII exhibited 1.44-fold higher ^1^O_2_ quantum yields than hematoporphyrin II in liposomes) [55] and modified eosins (their ^1^O_2_ quantum yields can differ by up to 1.82-fold) [56].

### 2.3. Designs of CPP-PS and CPP-Cargo-PS Conjugates

The use of CPP-cargo-PS conjugates provides an alternative PCI strategy for intracellular delivery of protein/peptide cargos, relying on more general PCI with a photosensitizer that is not chemically bound to the cargo. Examples of the structural designs of CPP-PS and CPP-cargo-PS conjugates used for PCI are illustrated in Figure 3.

When constructing CPP-cargo-PS for PCI, conjugation with the peptide/protein may affect the photochemical and photobiological properties of the photosensitizer. For instance, the hydrophobic photosensitizer Ce6 poorly localizes to endosomes, but it becomes amphipathic following conjugation with hydrophilic CPP, and the CPP-PS complex then localizes preferentially to endosomes, enabling PCI of CPP-PS [46]. Various photosensitizer candidate dyes linked with CPP-fusion protein (e.g., TatU1A) can localize to endocytic vesicles regardless of the dye’s own cellular localization. Thus, endosomal localization of conjugates is affected mainly by endocytic internalization of CPP-cargo-PS, which harbors a large peptide/protein moiety [35].

A number of CPP-PS and CPP-cargo-PS conjugates have been designed for use as PCI agents (Table 1). The mechanism of action, i.e., photoresponse of conjugates, may vary according to the design or structural characteristics of the conjugates. Notably, in the case of CPP-PS conjugates (specifically, conjugates of R9 peptide and 5(6)-carboxytetramethylrhodamine (TMR) fluorophore as a photosensitizer), neither N- nor C-terminally fluorophore-linked CPPs are as efficient as a construct in which a photosensitizer is attached to the middle of the CPP sequence for the photoinduced leakage of the membrane, indicating that the position where the photosensitizer is linked to the peptide affects the photolytic activity of conjugates [57].

CPP-cargo-PS conjugates for photo-dependent cytosolic RNA delivery have been developed and used by our group to study the PCI mechanism; TatU1A-PS conjugates have been constructed via covalent ligation between TatU1A protein bearing a C-terminal Cys residue and an organic photosensitizer with a maleimide group [35,58]. In this conjugate, Tat-fused U1A RNA-binding protein (RBP) was used as the protein moiety and would act as an RNA carrier. In this conjugate, U1A protein can be considered as a protein cargo when applied to the CPP-cargo-PS format, although RNAs are cargos of TatU1A–PS. Several CPP-RBP-PS variants with different CPP (Tat, flock house virus-derived peptide (FHV), and CTP512) and RBP (U1A, Sxl, and λN-peptide) combinations achieved different efficiencies of shRNA delivery and gene silencing [17].

It is known that other CPP-cargo-PS conjugates, in which the cargo is other than RBP, can be used for PCI. TatBim-Alexa546 consists of Tat peptide, Bim apoptosis-inducing peptide as the cargo, and Alexa546 as the photosensitizer (Figure 3b) [59]. This conjugate can mediate the photoinduction of apoptosis. Recently, this construct has been successfully used for the analysis of cell-cycle-dependent protein/peptide function via PCI-mediated peptide transduction [33].

The third example of CPP-cargo-PS is Tat-GFP-rhodamine, including GFP (green fluorescent protein) as the cargo [60]. In this conjugate, an average of three rhodamine groups were attached as photosensitizers per Tat–GFP molecule.

The addition of a hydrophobic amino acid linker between the photosensitizer and peptide/protein-enhanced PCI-mediated endosomal escape [53]. Altogether, these studies indicate that proper structural design of peptide-PS conjugates is essential for constructing a functional photosensitizing system.

## 3. Mechanism of PCI Using CPP-PS Conjugates

PCI relies on the photodynamic action of the photosensitizer and, specifically, ^1^O_2_ photogenerated from endocytosed photosensitizers, which then induces endosomal membrane permeabilization. Some lipids of the cellular membrane are vulnerable to photoinduced oxidation reactions in the presence of photosensitizers. Thus, the ^1^O_2_ photogeneration efficiency of a photosensitizer is important. In addition, direct contact between the photosensitizer and lipid is important for photoinduced membrane permeabilization [62]. Lipid photooxidation by the DO15 photosensitizer is much higher than that by methylene blue (MB), which has similar ^1^O_2_ generation efficiency but much less contact with the lipid membrane compared to that of DO15.

CPP-PS conjugates generally enter cells through the endocytosis pathway, in which cellular homeostasis regulates physiological parameters; hence, such parameters might play an important role in PCI of CPP-PS. Some reports have investigated the suitable physiological conditions for PCI and the changes in physiological conditions after PCI using CPP-PS. For example, PCI mediated by TAMRA-attached Tat peptide (TMR-Tat) leads to disruption of calcium homeostasis [63]. PCI-associated changes in intracellular calcium levels were also observed using TatU1A-Alexa546 after irradiation [64]. In addition, a low pH in the endosome is required for the photoinduced endosomal escape of TatU1A-Alexa546 [64].

Therefore, to understand the PCI mechanism, the following points should be discussed: (3.1) contribution of photophysical parameters of photosensitizers; (3.2) interaction of photogenerated ^1^O_2_ with the endosomal membrane; and (3.3~3.4) correlation between PCI and intracellular parameters, such as pH and Ca^2+^ concentration.

### 3.1. Contribution of Photophysical Parameters of Photosensitizers

**Figure 4:**
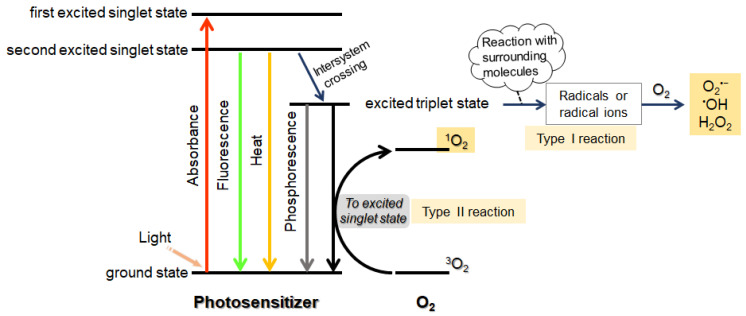
Photophysical and photochemical reactions triggered by photosensitizers.

To be suitable for PCI, photosensitizers should possess (1) a high absorption coefficient, (2) high quantum yield to generate ROS, and (3) high photostability [65]. After light absorption, photosensitizers are excited to the singlet state and, as they later decay back to the ground state, they emit fluorescence or heat. The excited photosensitizers can also undergo oxidation reactions triggered by interaction with molecular oxygen. This reaction can occur in two ways: type I and type II reactions (Figure 4) [66,67]. In type I reaction, photoinduced electron transfer from the excited photosensitizer to nearby substrates produces radical ions that can react with oxygen, leading to the formation of ROS, such as superoxide anion radicals (O_2_^−^), hydroxyl radicals (OH), and hydrogen peroxide (H_2_O_2_). In type II reaction, energy transfer from an excited photosensitizer in the triplet state to oxygen (^3^O_2_) generates ^1^O_2_. Most photosensitizers applied in PCI utilize type II reactions.

The relationship between photophysical parameters and PCI-mediated endosomal escape efficiency has been studied by our group [35]. Using TatU1A-PS conjugates with various photosensitizer moieties, we evaluated the photoinduced endosomal escape efficiency of TatU1A-PS/RNA complexes and measured fluorescence quantum yields, ^1^O_2_ quantum yields, and photoinduced heat generation efficiency of the photosensitizers. Photoinduced endosomal escape efficiency exhibited strong correlation only with ^1^O_2_ photogeneration efficiency.

Sufficient light energy is a basic requirement for photosensitizers to easily generate ROS. Therefore, light dose is key in determining the efficacy of PCI. In most clinical uses of photosensitizers for PDT, the effective light dose is around 100–200 J/cm^2^ [68]. In contrast, PCI generally requires a lower-light dose. PCI of the Tat-porphyrin conjugate with saporin required 5 min irradiation with blue light (7 mW/cm^2^, 2.1 J/cm^2^) [52]. PCI with TatU1A-Alexa546 or TatU1A-Alexa633 required irradiation at 20 J/cm^2^ [30]. Such a light dose causes very low cytotoxicity and allows for the repetitive delivery of cargos at different time points [31]. The inherent properties of each photosensitizer dictate the light energy required for PCI. This was demonstrated by our study, in which the same concentration of TatU1A-PS with various photosensitizer moieties required different light energy doses for PCI [30]. The amount of photosensitizer is also important to attain optimal cytosolic release of cargo [30,69]. In addition, a low-intensity long-duration PCI (0.05–0.2 mW/cm^2^ for 120 min) was reported to be more effective than standard acute PCI (2 mW/cm^2^ for 3–12 min) [70].

### 3.2. Interaction of Photogenerated ^1^O_2_ with the Endosomal Membrane

Endosomal localization of the photosensitizer is an important step in initiating ^1^O_2_-induced membrane destabilization. Photosensitizers with poor endosomal localization cannot effectively photodamage this organelle’s membrane even at high ^1^O_2_ quantum yield. This is because ^1^O_2_ has a short lifetime in the lipid bilayer (12–36 μs) [71] and even shorter (~4.2 μs) in aqueous medium [72], as well as limited diffusion (<20 nm) [73]. As a result, ^1^O_2_ -induced photodamage is highly localized [74]. Indeed, a major determinant of photoinduced cell death was found to be subcellular localization (to mitochondria) rather than the ^1^O_2_ quantum yield of photosensitizers [75]. The deeper the photosensitizer was in the membrane, the higher the photosensitizing efficiency achieved using porphyrin derivatives with hydrophobic modifications such as elongated alkyl carboxylate chains [42,76]. This phenomenon is probably due to the longer ^1^O_2_ lifetime in the lipid membrane than in aqueous medium. Similarly, adding a hydrophobic linker to the CPP-cargo-PS conjugate near the photosensitizer enhanced PCI efficiency [53], which may be due to improved lipophilicity of the moiety near the photosensitizer, allowing the photosensitizer to reach deeper in the lipid bilayer.

Reactivity between the membrane and light-activated photosensitizer relies largely on the interaction between ROS and membrane molecules. Biological membranes are composed of three types of lipids (phospholipids, glycolipids, and sterols) with membrane proteins and sugars regulating the structure and function of the membrane [77]. Phospholipids, proteins (especially at Tyr, Trp, His, Met, and Cys residues), and cholesterol are vulnerable to photooxidation by ^1^O_2_, whereas carbohydrate moieties of glycoproteins and glycolipids are less susceptible [78]. The composition of the endosomal membrane differs from that of the plasma membrane; for example, sphingomyelin and phosphatidylserine are more abundant, while diacyl phosphatidylcholine, diacyl phosphatidylethanolamine, and cholesterol are less abundant in the endosomal membrane [79,80]. It remains unknown which membrane components contribute most to PCI, although unsaturated phospholipids and cholesterol are known for being readily oxidized. Most studies on photooxidizable membrane mimetic models such as giant unilamellar vesicles showed that photooxidation led to an initial increase followed by a decrease in membrane surface, eventually causing membrane permeability due to hydrophobic defects or the generation of prepores [81,82]. Induction of this morphological change by photooxidation is related to the degree of lipid unsaturation in the membrane [83]. Membrane cholesterol might play a role in photoinduced endosomal membrane destabilization; shape transition and permeabilization of unsaturated lipid vesicles occurred after photooxidation but were delayed by cholesterol [84]. A decrease in cholesterol content in late endosomes may increase the vulnerability of the endosomal membrane to photosensitization and thus enable PCI.

In the case of CPP-mediated PCI, photooxidation of the endosomal membrane with photosensitizers is likely to be enhanced by binding of a cationic CPP to the anionic membrane surface. A study using liposomes showed that TMR-Tat bound to negatively charged liposomes but not to neutral ones and caused photo-dependent damage to the former [36]. CPPs may help destabilize the photooxidized endosomal membrane, as their accumulation may undermine the arrangement of membrane lipids [32].

### 3.3. Role of pH

Progressive acidification through endocytosis, from early endosomes (pH 6.0–6.5) to late endosomes (pH 5.5–6.0) and finally lysosomes (pH 4.5–5.5), is an important cellular event [85]. Endolysosomal trafficking and endosome escape of foreign molecules (such as pathogens, toxins, and viruses) are likely to depend on physiological conditions in endosomes, such as pH and concentration of calcium ions. Endosomal release of some viruses, such as flock house virus, has been suggested to require low endosomal pH [86]. SARS-CoV-2 and Ebola virus have been found to be related to endosomal pH and Ca^2+^ concentration [87,88]. Endosomal trafficking of these viruses is blocked by bafilomycin A1, which inhibits a proton pump (V-ATPase) that regulates endosomal acidity. Bafilomycin A1 inhibits also Ca^2+^ release from endosomes, suggesting an interplay between endosomal acidification and calcium loss from the endosome [89].

**Figure 5:**
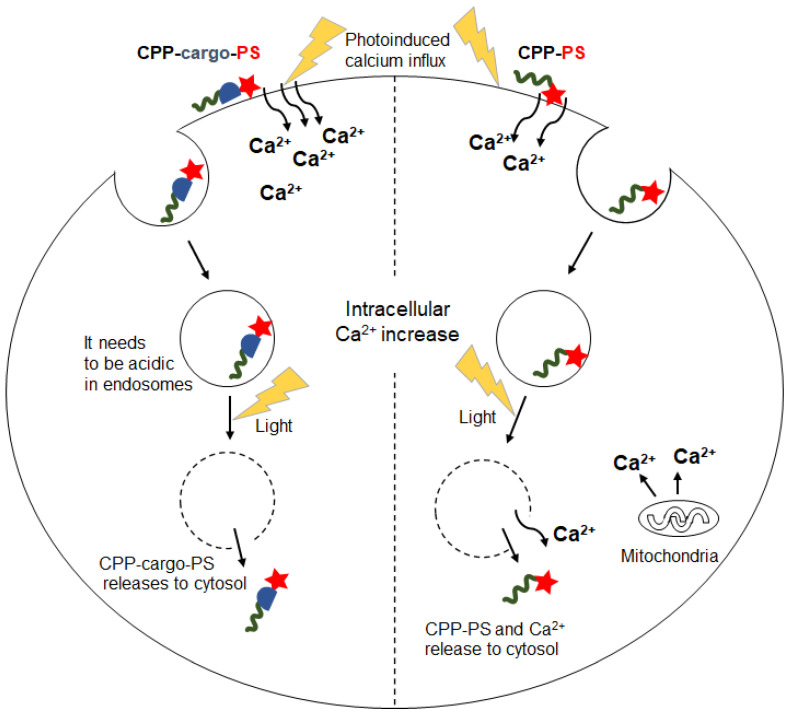
The role of intracellular parameters (Ca^2+^ and pH concentration) in CPP-cargo-PS- and CPP-PS-mediated PCI.

PCI-dependent endosomal release of CPP-fused molecules correlates with endosomal pH. PCI-mediated endosomal escape of the RNA carrier TatU1A–Alexa546 and shRNA accompanied an increase in intravesicular pH just before endosomal escape [35]. In contrast, elevation of endosomal pH by bafilomycin A1 or NH_4_Cl prevents photoinduced endosomal escape, indicating that endosomal acidification is necessary prior to photoirradiation (Figure 5) [64]. These results suggest that the elevated endosomal pH observed before endosomal escape is the result of photoinduced membrane destabilization, but not a prerequisite for PCI. Given that some CPP–protein constructs (TP/Arg_9_/Tat-biotin with avidin) were found to induce a population of nonacidic vesicles during trafficking through the endolysosomal pathway [90], the increase in endosomal pH may be due to photooxidation-triggered membrane destabilization assisted by the CPP.

### 3.4. Calcium Ion

Various amphipathic CPPs induce the influx of Ca^2+^, which can in turn activate membrane damage repair [91]. Ca^2+^ influx was caused by amphipathic CPPs, such as model amphipathic peptide (MAP) and transportan (TP), but not by cationic CPPs, such as Tat and Arg_9_. This is probably because the latter associate less stably with the plasma membrane and cannot interfere with membrane packing.

Several reports have described how photoirradiation of photosensitizer-treated cells induces an increase in cytoplasmic calcium ion concentration ([Ca^2+^]_i_) [92,93,94,95]. This photoinduced [Ca^2+^]_i_ increase is related to cellular damage and PDT, but is not desired for PCI. The source of the observed photoinduced [Ca^2+^]_i_ increase remains unclear, but is likely to occur through two mechanisms: one is the influx of calcium via impaired plasma membrane channels and the other is the release of ions from internal calcium-storage organelles, such as the ER, endosomes, and mitochondria. The interplay between ROS and Ca^2+^ in PDT has been reviewed recently [92,96]. Photoinduced intracellular Ca^2+^-related processes seem to depend on the localization of photosensitizers, including in the ER and mitochondria, which become photodamaged in PDT [94,95,97].

A photoinduced [Ca^2+^]_i_ increase has been reported in CPP-mediated PCI. TatU1A-PS, which is a CPP-cargo-PS construct, mediates the increase in [Ca^2+^]_i_ (Figure 5) [64]. This increase was abolished by using calcium-free medium, indicating that most calcium was imported from outside the cell instead of originating from internal calcium stores. Interestingly, the Ca^2+^ surge and endosomal escape of TatU1A-PS were independent phenomena [64], as the former but not the latter was blocked following photoinduction in a calcium-free medium. This is probably because the [Ca^2+^]_i_ increase is mediated by TatU1A-PS attached to the plasma membrane, whereas endosomal escape is mediated by TatU1A-PS localized in endosomes. Based on this finding, it may be possible to develop a PCI method with minimal side effects related to the [Ca^2+^]_i_ increase.

The photoinduced endosomal escape of TMR-Tat is accompanied by calcium release from the endosome [63]. The cytosolic calcium increase was slightly reduced in a calcium-free environment in TMR-Tat-mediated PCI, indicating that the surge originated in part from internal calcium stores and in part from extracellular influx. The different PCI outcomes regarding Ca^2+^ between TatU1A-PS and TMR-Tat [63,64] may be explained by differences in intracellular localization of these conjugates and/or membrane interaction mode. For example, when Tat is attached to the membrane, a photosensitizer directly connected to Tat seems to be forced to contact the membrane, but a photosensitizer in Tat–U1A-PS may be slightly off the membrane.

## 4. Future Perspectives and Limitations of CPP–PS Strategy

PCI using CPP-cargo-PS or CPP-PS with cargos can be used as a drug-delivery approach when the cargo is a drug. In the case of use of CPP-PS for cargo delivery, translating this approach into medicines has been limited by poor pharmacokinetic profiles, such as bioavailability, restricted organ distribution, and lack of target cell specificity of CPP, although the plasma half-lives of some photosensitizers (e.g., TPCS_2a_) are quite long and administration of TPCS_2a_ has been found to be safe in human trials [98,99,100]. The type of cargo seems to affect the pharmacokinetics and pharmacodynamics of CPP-cargo conjugates. For instance, tissue uptake for both Tat and cargo proteins is reduced when they are used as a conjugate [101]. In contrast, the conjugate of arginine-rich CPP and phosphorodiamidate morpholino oligomers (PMO) increases the elimination half-life and volume of distribution, and has greater tissue retention than the corresponding PMO [102]. CPP-PS-mediated PCI may not be applicable to some cells, in which the entry of CPPs is restricted, as the Tat-fluorescein conjugate cannot penetrate the intact plasma membrane of MDCK and CaCo2 cells [103,104,105].

As most CPPs do not have cell specificity, constructing an engineered CPP with targeting ligands or homing peptides for cancer has been one way to improve the usefulness of CPP in anticancer therapy [106]. The lack of a humoral immune response that has been reported for some CPPs is beneficial for clinical applications [107]. The photo-dependent spatiotemporal control of cytosolic molecular delivery using CPP-cargo-PS or CPP-PS is promising for studies on cell biology, such as cell polarization and early development.

## 5. Conclusions

This review highlights the structural designs of CPP(-cargo)-PS conjugates for PCI and the underlying mechanism. Many CPP-PS and CPP-cargo-PS conjugates have been designed as PCI agents, and have been successfully synthesized by bioconjugation techniques, such as ligation reactions via thiol- and amino-reactive photosensitizers. For efficient PCI with minimal side effects, the cells should be irradiated at an optimal light dose that depends on the photochemical activity of each CPP-PS, which is related mainly to the ^1^O_2_ quantum yield of the photosensitizer. Photoinduced endosomal escape is thought to derive from photooxidation of the endosomal membrane with photosensitizers. In the case of CPP-mediated PCI, membrane photooxidization is likely to be enhanced by binding of cationic CPPs to the anionic membrane surface. PCI mediated by several CPP-PS/CPP-cargo-PS systems accompanies increases in endosomal pH and cytosolic Ca^2+^. Whereas the source of increased Ca^2+^is still under debate, it probably depends on the localization of each CPP-PS/CPP-cargo-PS. Thus, proper design of CPP-PS/CPP-cargo-PS is essential for efficient endosomal escape and for avoiding unwanted side effects of PCI. CPP-PS-mediated PCI is a promising strategy for efficient macromolecular delivery, especially for cell biological studies. We believe this review provides the groundwork for understanding the molecular mechanism that enables efficient CPP-PS-mediated PCI.

## Figures and Tables

**Table 1:** CPP-PS and CPP-cargo-PS conjugates for PCI.

Conjugate	Response After Photoirradiation	^1^O_2_ Quantum Yield	PS at N or C Terminal	References
Tat–Ce6 Ce6–Tat	Enhanced delivery of toxin protein	High (0.62 and 0.69 for Ce6–Tat and Tat-Ce6, respectively, in CD_3_OD)	N/C	[43]
Tat–porphyrin porphyrin–Tat	Enhanced delivery of toxin protein	-	N/C	[52]
Tat–TPP *	Enhanced delivery of toxin protein	High (0.54 in CD_3_OD)	C	[61]
TatU1A–Alexa546 TatU1A–Alexa633 TatU1A–DY750 FHVU1A–Alexa546 TatSxl–Alexa546	Cytosolic delivery of RNAs and photoinduced RNAi	Very low (0.028 and 0.043 for Alexa546 and Alexa633, respectively, in octanol)	C	[17,30,35,58]
TatBim–Alexa546	Cytosolic delivery of Bim peptide and photoinduced apoptosis	-	C	[59]
Biotinylated TP10 + streptavidin–Alexa633	Cytosolic delivery of streptavidin–Alexa633	-	N	[32]

* 5,10,15,20-Tetraphenylporphine.
